# An inventory of native-alien populations in South Africa

**DOI:** 10.1038/s41597-023-02119-w

**Published:** 2023-04-15

**Authors:** Takalani Nelufule, Mark P. Robertson, John R. U. Wilson, Katelyn T. Faulkner

**Affiliations:** 1 Centre for Invasion Biology, Department of Zoology and Entomology, University of Pretoria, Pretoria, South Africa; 2 South African National Biodiversity Institute, Kirstenbosch Research Centre, Cape Town, South Africa; 3 Centre for Invasion Biology, Department of Botany and Zoology, Stellenbosch University, Stellenbosch, South Africa

**Keywords:** Zoology, Invasive species

## Abstract

Species can be both native and alien to a given administrative region. Here we present the first consolidated inventory of these ‘native-alien populations’ for South Africa, and provide an overview of the data it contains. To gather data, literature searches were performed and experts were consulted both directly and via an on-line survey. Putative native-alien populations were then scored based on a newly developed protocol. The final inventory contains information on 77 native species from 49 families across nine classes that have formed 132 native-alien populations across the terrestrial, freshwater, and marine environments. The phenomenon is rare when compared to the prevalence of related phenomena, such as alien species introduced from other countries (2033 alien species in South Africa), but is under-reported. However, they pose a specific problem for regulators and managers and their importance will likely increase with global change. These data will be integrated with an existing alien species list and, we hope, will provide a useful foundation to address the issue. We encourage those working on biodiversity to contribute more records.

## Background & Summary

The processes that lead to the introduction of alien species can act within political entities and, therefore, a species can be both native and alien within the same political entity^1–3^, a phenomenon for which the term ‘native-alien populations’ has been proposed^4^. This term was adapted from the term ‘native-alien species’ that is currently used in the Global Register of Introduced and Invasive Species, and by the IUCN SSC Invasive Species Specialist Group^5,6^. A native-alien population is defined as a population that results from the human-mediated dispersal of individuals of a species over a biogeographical boundary to a point beyond that species’ native range that is still within the same political entity as some parts of the species’ native range^4^. This definition differs slightly from others that are used to define this phenomenon in South Africa, and in other countries [e.g. ‘extralimital’ is used in Ellender and Weyl^7^ for fish in South Africa, and ‘domestic exotic’ is used in Guo and Ricklefs^1^ for plants in the United States of America (USA) and in Measey *et al*.^3^ for amphibians in South Africa]. The advantage of using the term native-alien populations is that it is explicit regarding the population’s status at national (native) and biogeographical (alien) levels, and as a protocol has been developed to implement this definition^8^. This means there is a process both to circumscribe the phenomenon and confirm instances, with a clear link through to the causes and consequences^9^.

Inventories of alien species have been compiled for many countries. Such inventories provide foundational data for research and policy, show the state of biodiversity, and inform the management of biological invasions (e.g. Pagad *et al*.^5^; Pauchard *et al*.^10^). However, few countries (e.g., Spain and USA) have included native-alien populations in these inventories^6^. This is concerning as native-alien populations pose specific regulatory and management challenges and tend to differ from alien populations introduced from other countries in the impacts caused^4^. This is, in part, as the regulation of biological invasions is often done at the country level, and consequently, native-alien populations are often regarded as native^11^. Similarly, in South Africa, there are only a few inventories that include native-alien populations: fish^7^, plants in the Garden Route National Park^12^, and amphibians^3^. Consequently, little information is available on how many and which native species have established native-alien populations^3^. This is despite these populations being recognised (as extralimital species) in the Alien and Invasive Species Regulations of South Africa’s National Environmental Management: Biodiversity Act of 2020 (henceforth NEM:BA: A&IS Regulations).

Under the NEM:BA A&IS Regulations, the South African National Biodiversity Institute is mandated to report on the status of biological invasions and their management every three years (see http://iasreport.sanbi.org.za/). As part of the process followed to produce the national status report on biological invasions, a South African alien species inventory has been developed to systematically and consistently record information in line with global data standards^13^. Such actions, and the inclusion of additional information on factors such as pathways of introduction and dispersal, date of introduction, and degree of establishment, increases the usefulness of these inventories^14^. Here we aimed to develop an inventory that: (1) consolidates the available information on native-alien populations in continental South Africa (i.e., excluding the sub-Antarctic Prince Edward Islands), (2) includes additional data on these populations that are vital for research and management; and (3) follows global data standards, and as such can be integrated with the existing alien species list produced as part of South Africa’s national status report on biological invasions. In this paper we present this inventory and provide an overview of the data it contains. This represents the first inventory of native-alien populations in South Africa, and is a step towards a greater understanding of native-alien populations and the biosecurity threat they pose.

## Methods

### Data collection

Records for native-alien populations in South Africa were gathered through an online survey, through direct discussions with experts, and through online searches.

An online survey was created using Google Forms (see Supplementary material 1), and experts were consulted through snowball sampling (experts were asked to nominate other experts until no new experts were identified). A request for information was also made during presentations at the South African National Symposium on Biological Invasions (Tulbagh 2019). Individuals who responded to this request were approached for information and were asked to nominate and provide the details of other experts who could provide information on this topic. Experts were then consulted via email and using the online survey between July 2020 and May 2021. This online survey included questions on the higher taxon-group, scientific name, common name, native range, location, and references for suspected native-alien populations (see Supplementary material 1 for full online survey and email that was sent to experts). A total of 21 of 29 experts contacted responded.

To augment the data obtained from the experts, the ISI Web of Knowledge and Google Scholar were used to search for scientific publications and grey literature on native-alien populations in South Africa. Searches were performed between February 2019 and May 2021. Terms that have been used to refer to this phenomenon (see Box 1 of Nelufule *et al*.^4^) were used as search strings, for example, “domestic exotics”, “intra-country established alien species” , “home-grown exotic” , “extralimital species” and “native-alien species” . Additional searches were performed by adding “AND South Africa” to these terms. Relevant papers (i.e. those with information on populations in South Africa) were selected based on the content of their titles and abstracts. A total of 23 published articles, two books and four scientific reports containing information on native-alien populations in South Africa were used for this study.

A draft inventory was produced using the collated information from the literature review and expert consultation. The draft inventory was sent back to the consulted experts for comments on errors and omissions six months after they were initially contacted. Follow-up emails were also sent to experts to encourage those who had not participated in the online survey to do so, and to encourage those who had participated to add any new suspected native-alien populations to the inventory. A request for individuals to consult the draft inventory and provide additional information (e.g. on populations missing from the inventory) was submitted to a South African list server on biological invasions on the 6^th^ of August 2020 (invasives@wordlink.co.za), at the time that the request was sent out 450 people subscribed to this list server. The online survey was also published in the newsletter of the Entomological Society of Southern Africa in August 2020^15^.

### Classification of native-alien populations

**Fig. 1:**
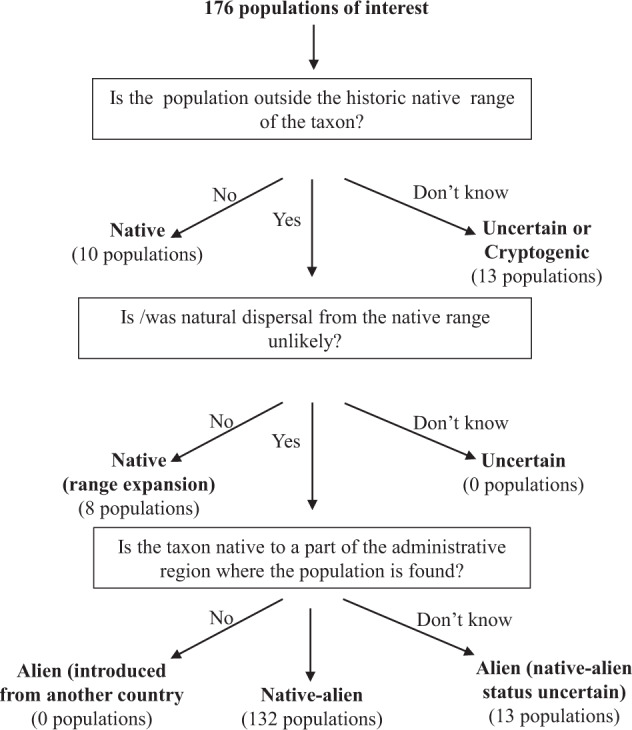
A flow diagram showing how many suspected populations were recorded and classified as native-alien populations in South Africa. These populations were classified using the newly developed protocol^8^.

The compiled inventory comprised a list of 176 suspected native-alien populations, of which 139 were collated from the literature and 37 from the experts (Fig. 1). The data collected on suspected native-alien populations came from various sources that used various frameworks and definitions. Therefore, a standardised protocol for classifying native-alien populations was developed and used to evaluate each population and determine whether it is a native, cryptogenic, alien or native-alien^8^. A total of 44 populations did not meet the criteria of a native-alien population during classification, and were excluded from the current inventory. Excluded populations either occurred within their native range (i.e. native populations), had uncertain native ranges (i.e. cryptogenic), were the result of range expansion in response to human-induced environmental change or there was uncertainty on whether the populations were outside their historic native range (Fig. 1).

### Structure of the inventory

We followed the data structure used for the species list of South Africa’s second national status report on biological invasions ‘The status of biological invasions and their management in South Africa in 2019’^13^. In line with this report we tried to ensure the data were FAIR (Findable, Accessible, Interoperable, and Reusable) and Darwin Core Terms were used where appropriate^16^ (see Table 1 for a list of terms used). The species names in the inventory were standardised according to the GBIF Backbone Taxonomy (10.15468/39omei, accessed: 20 May 2020). Dispersal pathways were classified using the classification scheme of the Convention on Biological Diversity^17^, following the guidance provided in Harrower *et al*.^18^. Introduction status was classified as per the unified framework for biological invasions^19^, environmental impacts were as per the Environmental Impact Classification for Alien Taxa scheme^20^, and socio-economic impacts as per the Socio-Economic Impact Classification for Alien Taxa scheme^21^. For each entry, we included the source from which the data were obtained and, following the guidance outlined in Wilson *et al*.^22^, a confidence estimate of high, medium, or low, was assigned so that the degree of confidence in every category is explicit (see the metadata for full explanations of these confidence estimates^23^).

**Table 1:** Summary of the information fields in the inventory “List of native-alien populations in South Africa”^23^.

Column name	Description	Values
scientificName	^*^The binomial name of the species or taxon including the authority as per Global Biodiversity Information Facility (GBIF) 10.15468/39omei accessed 20 May 2020.	Character
vernacularName	^*^The English name by which a species is known to the general public in South Africa.	Character
family	^*^The full scientific name of the family in which the taxon is classified.	Character
fingdom	^*^The full scientific name of the kingdom in which the taxon is classified.	Character
phylum	^*^The full scientific name of the phylum or division in which the taxon is classified.	Character
class	^*^The full scientific name of the class in which the taxon is classified.	Character
isNative	This specifies whether the species or taxon is native to South Africa or not.	Factor with three levels: Cryptogenic, TRUE, FALSE
occurrenceStatus	^*^This specifies whether the species or taxon has a native-alien population in South Africa.	Factor with four levels. Absent, Present, Doubtful, NotEvaluated
degreeOfEstablishment	^*^The coding as taken from the Unified Framework for Biological Invasions^19^, with the wording and description as per Groom *et al*.^16^.	Factor with 12 levels: A0, A1, B1, B2, B3, C0, C1, C2, C3, D1, D2, E
IntroductionStatus	A less refined measure of degree of establishment that consolidates several categories in degreeOfEstabishment	Factor with five levels: Casual; Established; Colonising; Invasive; WidespreadInvasive
pathway	^*^The process by which an organism came to be in a given place at a given time.	Factor with six major categories, and 44 sub-categories.
FirstRecord	The year the native-alien population was first recorded	Numeric
NativeRangeBroadAdmin	Provinces where the species occurs naturally in South Africa. This lists the occupancy of specific administrative regions in South Africa (the nine provinces for terrestrial systems; 22 water management areas for freshwater systems; and seven marine ecoregions).	Factor with 38 levels
AlienRangeBroadAdmin	Province where the species has formed population(s) outside its native range and outside of captivity or cultivation in South Africa. This lists the occupancy of specific administrative regions in South Africa (the nine provinces for terrestrial systems; 22 water management areas for freshwater systems; and seven marine ecoregions).	Factor with 38 levels
NativeRangeBroadEcol	Biomes where the species occurs as a native in South Africa.	Factor with eight levels: Fynbos, Nama-karoo, Albany-thicket, Savanna, Grassland, Indian Ocean Coastal Belt, Forest, Succulent-karoo biome
AlienRangeBroadEcol	Biomes where the species occurs as an alien in South Africa.	see NativeRangeBroadEcol
NativeRangeFreeText	Sites where the species occurs naturally.	Character
AlienRangeFree Text	Sites where the species has formed population(s) outside its native range and outside of captivity or cultivation.	Structured text field
impactEICAT Global	The maximum current recorded environmental impact anywhere in the world^20^,^32^. This is included here to give an indication of which taxa are known to cause damage.	Factor with seven levels: Minimal Concern, Minor, Moderate, Major, Massive, Not Evaluated, Data Deficient
impactSEICAT Global	The maximum current recorded socio-economic impact anywhere in the world^21^. This is included here to give an indication of which taxa are known to cause damage.	Factor with seven levels: Minimal Concern, Minor, Moderate, Major, Massive, Not Evaluated, Data Deficient

For each datum, we included a confidence estimate of high, medium, or low; the reference or source of the data; and comments or notes about the data collected. Included in the table are the column names of the inventory, a description of each column, and information on the values the data in each column can take. The column names are aligned with those of the species list of South Africa’s national status report on biological invasions (10.5281/zenodo.3947659), those marked by an asterisk in the description are also Darwin Core terms. In the inventory if a cell contains multiple values (e.g., taxa native to multiple provinces will have several values for NativeRangeBroadAdmin), values are separated by a pipe-delimiter, and in the case of dwc:pathway, categories and sub-categories are separated by a colon. For each column, NA is used if a value cannot confidently be ascribed or no value was found, noting that only taxa with valid scientific names that are known to be native to South Africa and have alien populations in the country are included.

## Data Records

### Structure of dataset

The dataset is archived and available from figshare.com as a comma delimited file (.csv)^23^. There are two files in figshare namely; “List of native-alien populations in South Africa.csv” , and “metadata.doc”. The dataset, contains an inventory of species native to South Africa that have formed native-alien populations in South Africa. The column names of the dataset are shown in Table 1. In the dataset each row below the header represents a record for a single native-alien population^23^. An NA in a cell means that no information was obtained, while DD means data deficient. The metadata contains a full description of the columns in the dataset^23^.

### Summary of the inventory

We found a total of 77 native species from 49 families across nine classes that have formed 132 native-alien populations in South Africa (Tables 2–4). A total of 109 populations were identified through the literature search, while 23 populations were identified through expert consultation. Three of the recorded native species with native-alien populations are listed under the NEMBA A&IS Regulations 2020: *Clarias gariepinus* (African sharptooth catfish); *Hyperolius marmoratus* (painted reed frog); and *Sclerophrys gutturalis* (guttural toad).

**Table 2:** South African native plant species that have formed native-alien populations in South Africa, with selected information taken from the inventory “List of native-alien populations in South Africa”.

scientificName	family	pathway	Reference
*Brachylaena discolor* DC.	Asteraceae	Escape	T. Rebelo 2020: Pers.comm
*Carissa macrocarpa* (Eckl.) A.DC.	Apocynaceae	Escape	J. Baard 2020: Pers.com
*Crassula multicava* Lem.	Crassulaceae	Escape	^33^
*Cyperus papyrus* L.	Cyperaceae	Escape	^33^
*Dais cotinifolia* L.	Thymelaeaceae	Escape	^33^
*Ekebergia capensis* Sparrm.	Meliaceae	Escape	T. Rebelo 2020: Pers.comm
*Erythrina* cf. *lysistemon* Hutch.	Fabaceae	Escape	^33^
*Euryops virgineus* (L.f.) DC.	Asteraceae	Escape	T. Rebelo 2020: Pers.comm
*Gynandropsis gynandra* L.	Cleomaceae	Escape	^34^
*Harpephyllum caffrum* Bernh.	Anacardiaceae	Escape	T. Rebelo 2020: Pers.comm
*Ipomoea cairica* (L.) Sweet	Convolvulaceae	Escape	^33^
*Jasminum multipartitum* Hochst.	Oleaceae	Escape	T. Jaca 2020: Pers.comm
*Podocarpus henkelii* Stapf ex Dallim. and Jacks.	Podocarpaceae	Escape	^33^
*Rauvolfia caffra* Sond.	Apocynaceae	Escape	T. Rebelo 2020: Pers.comm
*Senecio angulatus* L.fil.	Asteraceae	Escape	T. Rebelo 2020: Pers.comm
*Setaria megaphylla* (Steud.) T.Durand & Schinz	Poaceae	Escape	N. Joubert 2020: Pers.comm
*Syzygium cordatum* Hochst. ex Krauss	Myrtaceae	Escape	N. Joubert 2020: Pers.comm
*Tecoma capensis* (Thunb.) Lindl.	Bignoniaceae	Escape	^33^
*Tetradenia riparia* (Hochst.) Codd	Lamiaceae	Escape	J. Baard 2020: Pers.comm
*Thunbergia alata* Bojer ex Sims	Acanthaceae	Escape	^33^

Only high level pathway categories are displayed here, for sub-categories see the full database. Note this table includes one row per taxon/species whereas the full database has one row per population. The full database is available: 10.6084/m9.figshare.21084829.v19.

**Table 3:** South African native vertebrate species that have formed native-alien populations in South Africa, with selected information taken from the inventory “List of native-alien populations in South Africa”.

scientificName	family	pathway	Reference
*Acinonyx jubatus* (Von Schreber, 1775)	Felidae	Release	^25^
*Aepyceros melampus* (Lichtenstein, 1812)	Bovidae	Release	^25^
*Agapornis roseicollis* (Vieillot, 1818)	Psittacidae	Escape	^35^
*Bradypodion ventrale* (Gray, 1845)	Chamaeleonidae	Release	^36^
*Ceratotherium simum* (Burchell, 1817)	Rhinocerotidae	Release	^25^
*Chetia brevis* Jubb, 1968	Cichlidae	Release	^37^
*Clarias gariepinus* (Burchell, 1822)	Clariidae	Release| Escape| Corridor	^37^
*Connochaetes gnou* (Zimmermann, 1780)	Bovidae	Release	^38^
*Connochaetes taurinus* (Burchell, 1823)	Bovidae	Release	^38^
*Coptodon* *sparrmanii* Smith, 1840	Cichlidae	Release	^37^
*Damaliscus pygargus phillipsi* Harper, 1939	Bovidae	Release	^25^
*Damaliscus pygargus pygargus* Pallas, 1767	Bovidae	Release	^24^
*Enteromius anoplus* Weber, 1897	Cyprinidae	Escape	^37^
*Enteromius treurensis* (Groenewald, 1958)	Cyprinidae	Release	^37^
*Equus quagga* Boddaert, 1785	Equidae	Release	^38^
*Giraffa camelopardalis* (Linnaeus, 1758)	Giraffidae	Release	^25^
*Hemidactylus mabouia* (Moreau De Jonnès, 1818)	Gekkonidae	Stowaway	^39^
*Hippotragus niger* (Harris, 1838)	Bovidae	Release	^24^
*Hyperolius marmoratus* Rapp, 1842	Hyperoliidae	Contaminant	^3^
*Kneria auriculata* (Pellegrin, 1905)	Kneriidae	Release	^37^
*Kobus ellipsiprymnus* (Ogilby, 1833)	Bovidae	Release	^24^
*Labeo capensis* (Smith, 1841)	Cyprinidae	Release| Corridor	^37^
*Labeo umbratus* (Smith, 1841)	Cyprinidae	Release	^37^
*Labeobarbus aeneus* (Burchell, 1822)	Cyprinidae	Release| Corridor	^37^
*Labeobarbus capensis* (Smith, 1841)	Cyprinidae	Corridor	^37^
*Lonchura fringilloides* (Lafresnaye, 1835)	Estrildidae	Escape	I. Little 2020: Pers.comm
*Lygodactylus capensis* (Smith, 1849)	Chamaeleonidae	Stowaway	^39^
*Nothobranchius rachovii* Ahl, 1926	Nothobranchiidae	Release	^37^
*Numida meleagris* (Linnaeus, 1758)	Numididae	Release	^40^
*Oreochromis mossambicus* (Peters, 1852)	Cichlidae	Escape	^37^
*Redunca fulvorufula* (Afzelius, 1815)	Bovidae	Release	^24^
*Sclerophrys gutturalis* (Power, 1927)	Bufonidae	Contaminant	^3^
*Tilapia rendalli* (Boulenger, 1897)	Cichlidae	Release	^37^
*Tragelaphus angasii* Angas, 1849	Bovidae	Release	^41,42^

Only high level pathway categories are displayed here, for sub-categories see the full database. Note this table includes one row per taxon/species whereas the full database has one row per population. The full database is available: 10.6084/m9.figshare.21084829.v19.

**Table 4:** South African native invertebrate species that have formed native-alien populations in South Africa, with selected information taken from the inventory “List of native-alien populations in South Africa”. Only high level pathway categories are displayed here, for sub-categories see the full database. Note this table includes one row per taxon/species whereas the full database has one row per population. The full database is available: 10.6084/m9.figshare.21084829.v19.

scientificName	family	pathway	Reference
*Anisorrhina flavomaculata* (Fabricius, 1798)	Scarabaeidae	Contaminant	^43^
*Atoxonoides meridionalis* (Forcart, 1967)	Urocyclidae	Contaminant	^44^
*Austruca occidentalis* (Naderloo, Schubart & H.-T. Shih, 2016)	Ocypodidae	Unknown	^45^
*Charaxes brutus natalensis* Staudinger, 1886	Nymphalidae	Contaminant	^46^
*Chlorocala Africana* *subsuturalis* (Kraatz, 1891)	Scarabaeidae	Contaminant	^43^
*Cochlitoma zebra* (Bruguière, 1792)	Achatinidae	Release	^44^
*Cochlochlila bullita* (Stål, 1873)	Tingidae	Contaminant	^47^
*Coeliades libeon* (Druce, 1875)	Hesperiidae	Contaminant	^46^
*Dicronorhina derbyana subsp. derbyana* Westwood, 1842	Scarabaeidae	Contaminant	^43^
*Ellimenistes laesicollis* Fåhraeus 1871	Curculionidae	Contaminant	^48^
*Glutophrissa sabina* (Felder & Felder, 1865)	Pieridae	Contaminant	^46^
*Haliotis midae* Linnaeus, 1758	Haliotidae	Escape	G. Branch 2020: pers.comm
*Junonia orithya madagascariensis* Guenée, 1872	Nymphalidae	Contaminant	^46^
*Laevicaulis alte* (Férussac, 1822)	Veronicellidae	Contaminant	^44^
*Leucocelis rubra* (Gory & Percheron, 1833)	Scarabaeidae	Contaminant	^46^
*Mausoleopsis amabilis* (Schaum, 1844)	Scarabaeidae	Contaminant	^43^
*Mylothris agathina* (Cramer, 1779)	Pieridae	Contaminant	^46^
*Nata vernicosa* (F.Krauss, 1848)	Rhytididae	Contaminant	^49^
*Neuranethes spodopterodes Hampson, 1908*	Noctuidae	Contaminant	^50^
*Ocypode ceratophthalmus(* Pallas, 1772)	Ocypodidae	Unknown	^45^
*Pachnoda sinuata flaviventris* (Gory & Percheron, 1833)	Scarabaeidae	Contaminant	^43^
*Portunus segnis* (Forskål, 1775)	Portunidae	Other release	^45^
*Varuna litterata* (Fabricius, 1798)	Varunidae	Unknown	^45^

**Fig. 2:**
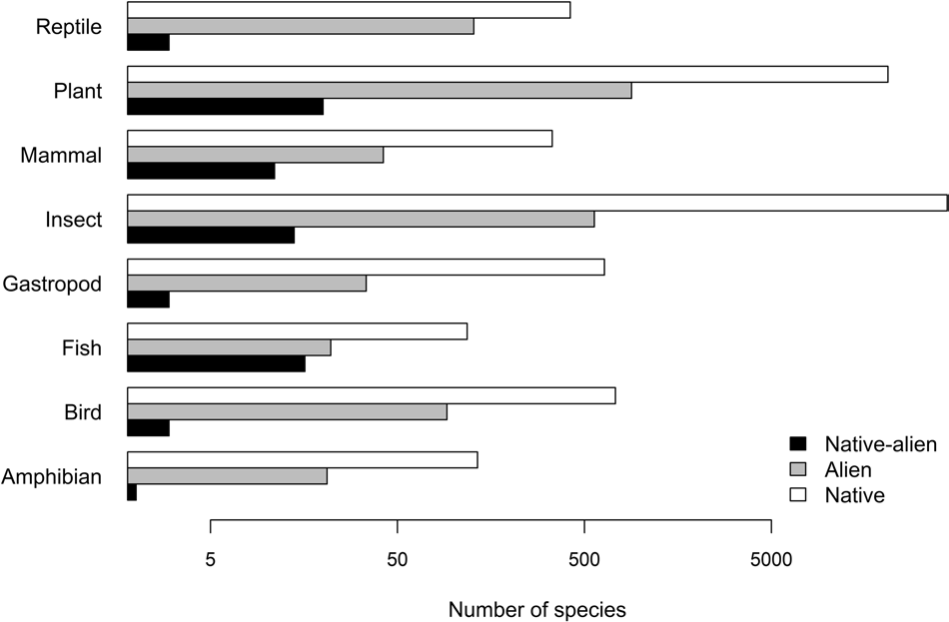
The number of native species with native-alien populations (black), the number of alien species introduced to South Africa from other countries (grey), and number of native species from South Africa (white) across eight taxonomic groups. Note that the axis is on a log scale. Native-alien population data are from this study, data on other alien species are from the species list of South Africa’s national status report on biological invasions^13^, and native species data are from the National Biodiversity Assessment^51^.

Native-alien populations are rare when compared to the prevalence of related phenomena—0.1% of native species have formed native-alien populations, and the number of alien species introduced from other countries is 25-fold higher than the number of species with native-alien populations—but it is likely under-reported. Native-alien populations are particularly prevalent in specific taxonomic groups. Most species with native-alien populations were plants, and plants had more recorded native-alien populations than other taxonomic groups. However, fish had the highest percentage of native species with recorded native-alien populations (Fig. 2). All other taxa had a low percentage of native species with recorded native-alien populations (<= 4%) (Supplementary Fig. 1). The terrestrial environment (n = 101) had a higher number of recorded native-alien populations than freshwater (n = 26) or marine (n = 5) environments .

**Fig. 3:**
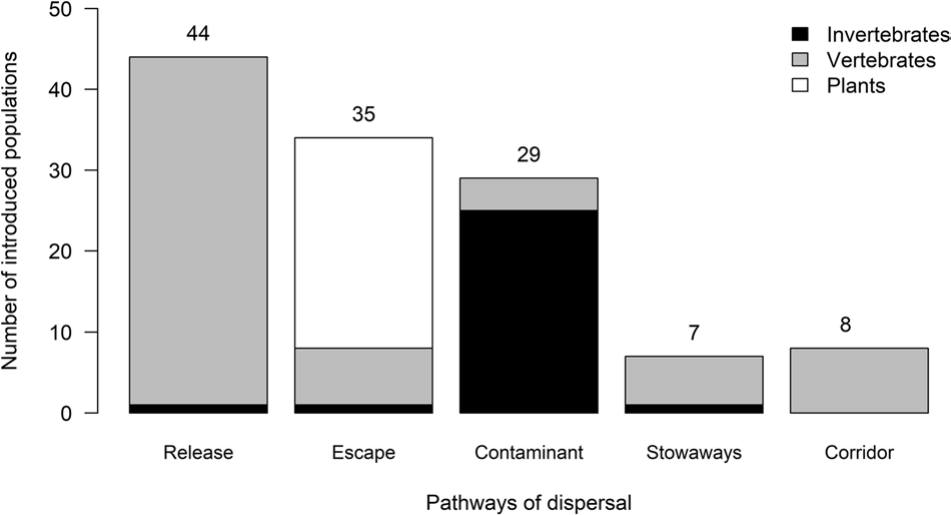
The number of terrestrial vertebrate, invertebrate and plant native-alien populations moved within South Africa through the pathways of dispersal (main categories of the CBD pathway framework^17^). The numbers above the bar graph are the total number of introduced native-alien populations per pathway, excluding the populations for which pathway(s) of dispersal were unknown (invertebrate, n = 5; vertebrates, n = 2; plants, n = 1).

**Fig. 4:**
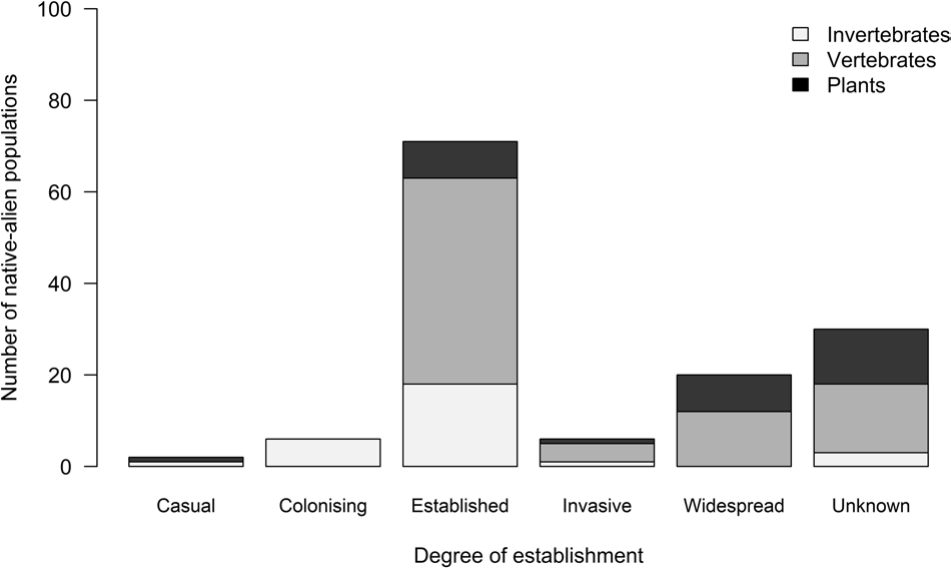
The degree of establishment of vertebrate, invertebrate and plant native-alien populations in South Africa.

The pathways of dispersal through which these populations were introduced differed by organism type (Fig. 3). Most fish and mammal populations were released intentionally, while most bird and plant populations escaped from captivity or cultivation. For many groups, the majority of the populations were accidentally introduced, with most gastropod, amphibian and insect populations introduced as contaminants on transported goods, and most reptile populations introduced as stowaways on transport vehicles. Mammals were released for hunting and improving eco-tourism^24,25^. Some individuals of native-alien populations were dispersed through a single pathway while others were dispersed through more than one pathway of dispersal. All species that dispersed through corridors were fish (Fig. 3). Data for degree of establishment was available for 77% of the recorded native-alien populations, but a relatively large number of vertebrate and plant native-alien populations had an unknown degree of establishment (Fig. 4). The majority of native-alien populations (59%) are established, while very few (18%) are invasive (Fig. 4).

There were few records of potential impacts, but for the populations for which they were available, these impacts differ between organism types and were mostly Minor^23^. The highest impact recorded for gastropods and fish was Minor, Moderate for plants, amphibians, insects, and mammals, and Major for reptiles. *Hemidactylus mabouia* (Tropical house gecko) was recorded as having Major environmental impacts when introduced to another country. The gecko competes with native species for resources in the United States of America (USA) where it has caused the loss of local populations^26^.

The majority of species with native-alien populations (74%) are native to multiple provinces and biomes of South Africa, while 24% are native to one province and biome^23^.

**Fig. 5:**
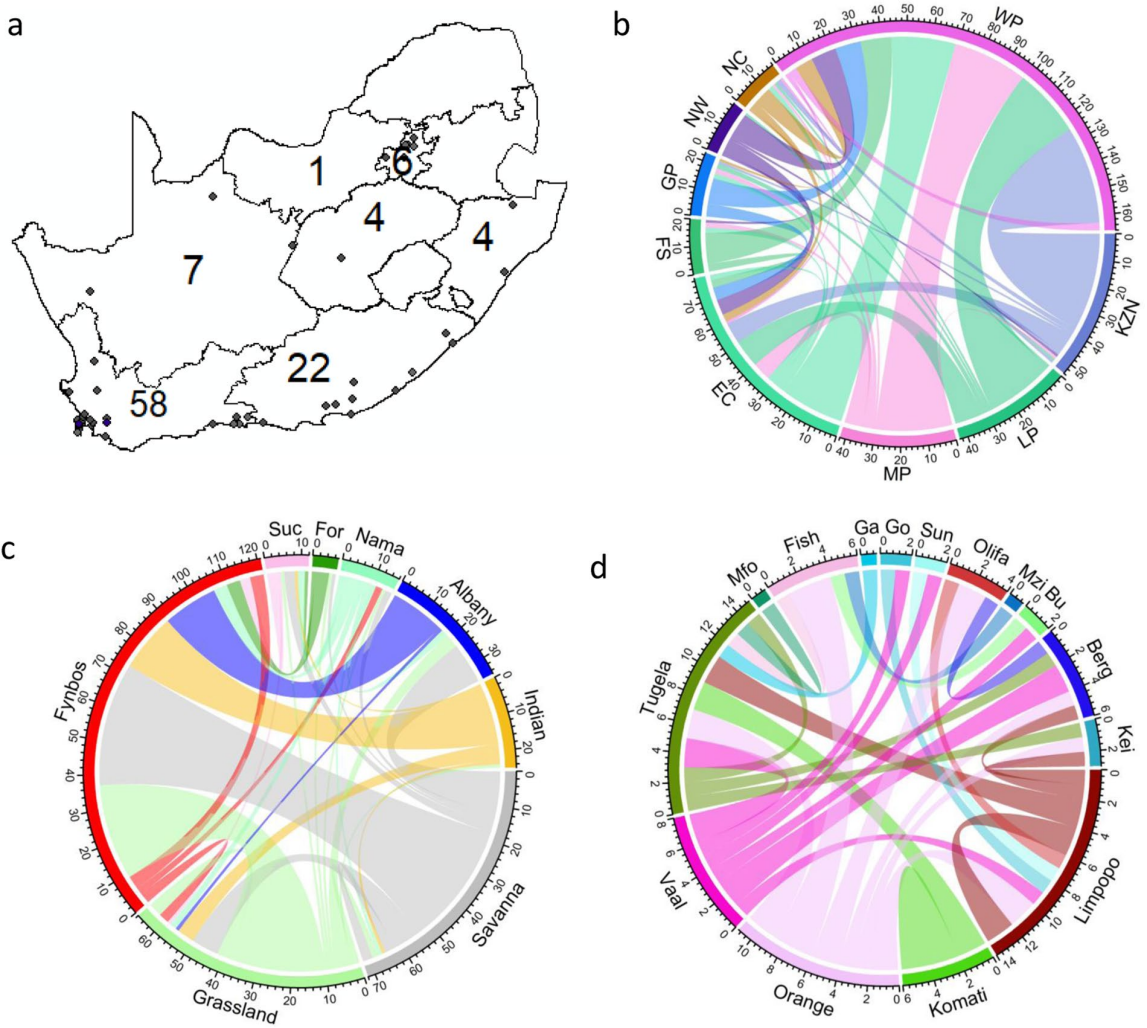
(**a**) A map of the location of native-alien populations for which precise information on location was available; and the exchange of native-alien populations between: (**b**) the provinces; (**c**) the biomes; and (**d**) the river catchments of South Africa. The coloured lines indicate the opposite flow (native species to provinces, biomes and river catchments where they have formed native-alien populations). Each tick on the outside of the plot corresponds to one population and the thickness of the lines is proportional to the total number of populations. Populations were excluded if the origin location within the native range was uncertain. Precise information on location was only available for 59 native-alien populations. The distribution of native-alien populations in South Africa was mapped using ArcGis^52^ (ESRI 2020). The circlize package in R^53^ (Gu 2014) was used to draw chord diagrams. Fynbos = Fynbos biome; Suc = Succulent-Karoo biome; Albany = Albany Thicket biome; For = Forest biome; Indian = Indian Coastal Belt biome; Grassland = Grassland biome; Nama = Nama-Karoo biome; Savanna = Savanna biome. Mfo = Mfolozi river catchment, Olifa = Olifants river catchment; Berg = Berg river catchment; Orange = Orange river catchment; Vaal = Vaal river catchment; Tugela = Tugela river catchment; Fish = Fish river catchment; Sun = Sundays river catchment; Bu = Bushman’s river catchment; Kei = Keiskamma river catchment; Go = Gouritz river catchment; Mzi = Mzimvubu river catchment; Ga = Gamtoos river catchment. WP = Western Cape province, EC = Eastern Cape province, NC = Northern Cape province, GP = Gauteng province, FS = Free State province, KZN = KwaZulu-Natal province, LP = Limpopo province.

South African native species have formed native-alien populations across the terrestrial, freshwater, and marine environments, with native-alien populations more prevalent in some parts of South Africa than others (Fig 5a).

The Western Cape province (n = 58) had the highest number of recorded terrestrial native-alien populations followed by the Eastern Cape province (n = 22) (Fig. 5a). All other provinces had few recorded terrestrial native-alien populations (n < = 7) (Fig. 5a). Most of the native-alien populations that have been introduced to the Western Cape province are native to the KwaZulu-Natal province followed by the Limpopo, Mpumalanga and Eastern Cape provinces (Fig. 5b).

The Fynbos biome (n = 55) had the highest number of recorded terrestrial native-alien populations followed by the Grassland (n = 19), and Albany Thicket biomes (n = 11) (Supplementary Fig. 2). All other biomes had few recorded terrestrial native-alien populations (n < = 8) (Supplementary Fig. 2). Most of the native-alien populations that have been introduced to the Fynbos biome are native to the Savanna and Grassland biomes (Fig. 5c).

Of the biomes, the Fynbos had the greatest number of recorded native-alien populations. The Fynbos (0.06 native-alien populations per km^2^) and Albany Thicket biomes (0.04 native-alien populations per km^2^) had a relatively large number of recorded native-alien populations relative to their total area. The Forest (0.01 native-alien populations per km^2^), Succulent-Karoo (0.01 native-alien populations per km^2^), Savanna (<0.01 native-alien populations per km^2^), Nama-Karoo (<0.01 native-alien populations per km^2^), and Grassland biomes (<0.01 native-alien populations per km^2^) had the lowest number of recorded native-alien populations relative to their total area.

The Tugela river catchment (n = 12) had the highest number of recorded freshwater native-alien populations followed by the Limpopo river catchment (n = 8) (Supplementary Fig. 3). All other river catchments had few recorded freshwater native-alien populations (n < 6) (Supplementary Fig. 3). The majority of freshwater fish native-alien populations are native to the Orange and Vaal river catchments (Fig. 5d).

The Agulhas ecoregion (n = 4) had the highest number of recorded marine native-alien populations followed by the Benguela ecoregion (n = 1)^23^. The majority of marine native-alien populations are native to the Delagoa and Natal ecoregions^23^.

## Technical Validation

### Record verification

Records were collected, the taxonomy was standardised, where possible, using the GBIF taxonomic backbone, and sent to a taxonomist for a further check. Experts from different fields also confirmed the existence of the records from the online inventory. If any information recorded in the inventory was unclear and could not be verified by the authors, the record of the population was traced back to the original manuscript. All records that could not be verified were excluded from the inventory.

## Usage Notes

The goal of this inventory is to ensure that native-alien populations are correctly classified, separated from other alien populations, included in alien species inventories, and confirmed following a standardised framework. The inventory could be used as a template to assist countries to collate information on within-country invasions that follow global data standards. The collated data can be used to report on the state of biological invasions, and inform the monitoring, and management of these invasions, and is required to track progress towards biodiversity targets (e.g. the Convention on Biological Diversity’s Aichi Target 9 (www.cbd.int/sp/targets/), and the post-2020 targets^27^). As the taxonomic names in the inventory were standardised using the GBIF taxonomic backbone, the data from it can be easily integrated with the existing alien species list produced as part of South Africa’s national status report on biological invasions. For further information Takalani Nelufule can be contacted through email at: takalani.nelu@gmail.com.

### Limitations

We found several limitations when creating the inventory of native-alien populations in South Africa, for example, the lack of a standardised protocol for collecting information on these populations, lack of precise location data for the native range and for native-alien populations, and a lack of information on the biogeographical barrier that separates native and native-alien populations. The experts who collected the records of native-alien populations did not follow a standardised protocol or definition, and a clear description of the precise location (e.g. distance and direction from nearest town or a coordinate of the centroid of the population) of the native range and native-alien population were not available in most cases. Without information on the precise location of the native-alien population it will be difficult for researchers and managers to find these populations. Information on the biogeographical barriers that separate the native-alien population from its native range, the date of introduction, and impact, were also not available for the majority of the native-alien populations. This information is vital for the management of these biological invasions and is useful for the prediction of future trends. To overcome these limitations, and improve the understanding of, and quality of data for, native-alien populations, protocols and global data standards were implemented when compiling the inventory presented here. We suggest that these protocols and data standards be used in future to create lists of native-alien populations to allow for comparisons across taxa and regions. There are several classes for which no native-alien populations were recorded, including Arachnida, Chilopoda, Crustacea, and Annelida. It is likely, however, that for some of these taxa native-alien populations do exist in South Africa, but have not been recorded as invertebrates are generally understudied^28^. In addition, it is likely that many native-alien populations may not have been reported as the phenomenon has not been well described and its importance has not been fully appreciated^29^. For example, some native Arachnid and Annelid species are offered for sale in the pet trade in South Africa^30^^,^^31^ and could have formed native-alien populations by being released irresponsibly in areas beyond their native ranges. These groups require research attention if we are to better understand the scope of the native-alien population phenomenon in South Africa.

## Supplementary information

An inventory of native-alien population populations

## Data Availability

No custom computer code or algorithms were used to process or generate the data presented in this manuscript.
