# Publisher Correction: An inventory of native-alien populations in South Africa

**DOI:** 10.1038/s41597-023-02216-w

**Published:** 2023-05-18

**Authors:** Takalani Nelufule, Mark P. Robertson, John R. U. Wilson, Katelyn T. Faulkner

**Affiliations:** 1 Centre for Invasion Biology, Department of Zoology and Entomology, University of Pretoria, Pretoria, South Africa; 2 South African National Biodiversity Institute, Kirstenbosch Research Centre, Cape Town, South Africa; 3 Centre for Invasion Biology, Department of Botany and Zoology, Stellenbosch University, Stellenbosch, South Africa

**Keywords:** Zoology, Invasive species

Correction to: *Scientific Data* 10.1038/s41597-023-02119-w, published online 15 April 2023

The original manuscript contained errors in the reference list order, meaning some reference numbers pointed to the wrong citation. The affected references numbers are 28–32, 36, and 52-53.

This has been corrected in the HTML and PDF versions of the manuscript. No references were added or removed, just re-ordered vs their original reference numbers.

