# Supplementary material for: An inventory of native-alien populations in South Africa
Source: Sci Data. 2023 Apr 15;10:213. doi: 10.1038/s41597-023-02119-w (PMC10105770; doi:10.1038/s41597-023-02119-w)
Supplement: Supplementary file 1 — An inventory of native-alien population populations [file 41597_2023_2119_MOESM1_ESM.docx]

**An inventory of native-alien populations in South Africa**

Takalani Nelufule^1,2^*, Mark P. Robertson^1^, John R.U. Wilson^2,3^ and Katelyn T. Faulkner^1,2^

^1^Centre for Invasion Biology, Department of Zoology and Entomology, University of Pretoria, Pretoria, South Africa

^2^South African National Biodiversity Institute, Kirstenbosch Research Centre, Cape Town, South Africa

^3^Centre for Invasion Biology, Department of Botany and Zoology, Stellenbosch University, Stellenbosch, South Africa

* Corresponding Author: Takalani Nelufule

Email: takalani.nelu@gmail.com

Tel: +27 (0) 679071346

ORCID: <http://orcid.org/0000-0001-9823-075X>

Other emails: mrobertson@zoology.up.ac.za (ORCID: https://orcid.org/0000-0003-3225-6302); john.wilson2@gmail.com (ORCID: https://orcid.org/0000-0003-0174-3239); katelynfaulkner@gmail.com (ORCID: <https://orcid.org/0000-0002-3955-353X>)

**Supplementary 1 Online survey**

Dear Sir/Madam

I would hereby like to invite you to participate (edit and/or add new populations) in an online inventory for which a link is provided below

The aim of the inventory is to determine species that are native to one part of South Africa that have formed alien populations elsewhere in the country. These are the species that have been moved (presumably by humans) from one site to another, and established populations that seem to be self-sustaining. These species are known as extralimital species or domestic exotics. This survey exclude species that have expanded their range in response to human modification of the environment (e.g. *haededas*), or those that have locally increased in density (e.g. bush encroachment). This study is part of a Doctor of Philosophy (PhD) study at the University of Pretoria in collaboration with the South African National Biodiversity Institute through funding from the Department of Forestry, Fisheries and the Environment. We thank you for your participation.

This survey is voluntary and your participation will be very much appreciated. Thank you.

Should you wish to obtain more information about this study, please feel free to contact me at: [takalani.nelu@gmail.com](mailto:takalani.nelu@gmail.com) or Prof Mark Robertson at: [mrobertson@zoology.up.ac.za](mailto:mrobertson@zoology.up.ac.za)

Please follow the link below:

<https://docs.google.com/spreadsheets/d/1bhxvRbWeY-lVcgfI-1ybVA8aj4aqZudTztP05xQg-pU/edit?usp=sharing>

***Follow-up***

Dear Sir/Madam

As part of my PhD research I have been collecting data on species that are native to one part of South Africa and that have formed alien populations elsewhere in the country. These are species that have been moved (presumably by humans) from one site to another, and established populations that seem to be self-sustaining. These species are known as extralimital species or domestic exotics. They exclude species that have expanded their range in response to human modification of the environment (e.g. hadedas), or those that have locally increased in density (e.g. bush encroachment). While these species are native to the country, they can have negative impacts where they are introduced. These species have received little research attention, and with my research I hope to improve our knowledge on these introductions and their impacts.

I previously contacted you regarding this work in the hope that you may have information that you are willing to share on such species. Unfortunately, I have collected very few records for marine, vertebrates and invertebrates (see attached for the preliminary results), and so I am following up on that previous email.

Please let me know if you do have any information you are willing to share. I can be contacted at takalani.nelu@gmail.com. Alternatively, you can follow the link below to edit or add species on the online inventory. Any information you can provide would be greatly appreciated.

https://docs.google.com/spreadsheets/d/1bhxvRbWeY-lVcgfI-1ybVA8aj4aqZudTztP05xQg-pU/edit?usp=sharing

Should you wish to obtain more information about this study, please feel free to contact me or my supervisor, Prof Mark Robertson (mrobertson@zoology.up.ac.za).

Kind regards

Takalani

**Supplementary Figure 1**: Numbers of native species with recorded native-alien populations in particular taxonomic groups in South Africa (n = 77) relative to the total number of native species in South Africa per taxonomic group (both axes have been logged). The taxonomic groups below the line have fewer species with recorded native-alien populations than would be expected from the total number of native species.


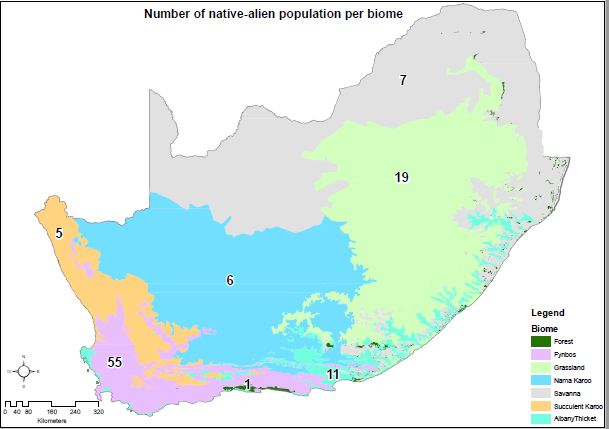


**Supplementary Figure 2**: Number of recorded terrestrial native-alien populations across different biomes of South Africa.


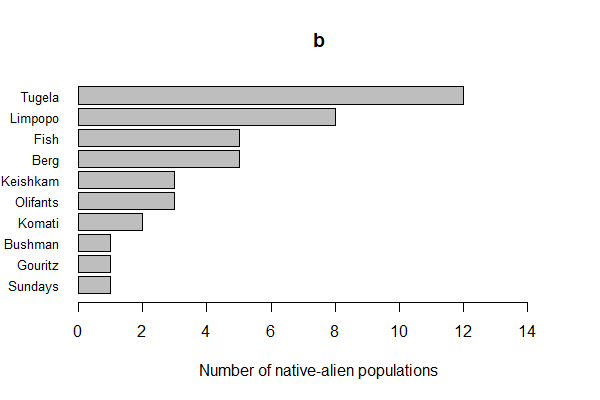


**Supplementary Figure 3**: Number of recorded freshwater native-alien populations across different catchment areas of South Africa. Olifants = Olifants river catchment; Komati = Inkomati river catchment; Limpopo = Limpopo river catchment, Berg = Berg river catchment; Tugela = Tugela river catchment; Fish = Fish river catchment; Sundays = Sundays river catchment; Bushman = Bushman’s river catchment; Keishkam = Keiskamma river catchment; Gouritz = Gouritz river catchment.
